# The first complete chloroplast genome of *Halodule uninervis* (Forssk.) Boiss. 1882 (Cymodoceaceae)

**DOI:** 10.1080/23802359.2024.2429635

**Published:** 2024-11-20

**Authors:** Mingzhong Liu, Rongrong Shan, Jiaxin Wu, Yunfeng Shi, Muqiu Zhao

**Affiliations:** Key Laboratory for Coastal Marine Eco-Environment Process and Carbon Sink of Hainan province, Yazhou Bay Innovation Institute, College of Ecology and Environment, Hainan Tropical Ocean University, Sanya, China

**Keywords:** *Halodule uninervis*, Cymodoceaceae, chloroplast genome, next-generation sequencing, phylogenetic analysis

## Abstract

*Halodule uninervis* (Forssk.) Boiss. 1882 is a seagrass species. The complete chloroplast genome is 164,005 bp in length, with a GC content of 36.3%. This genome consists of a large single-copy region of 87,522 bp, a small single-copy region of 11,481 bp and a pair of inverse repeat regions of 32,501 bp each. A total of 131 genes were annotated in the genome, including 87 protein-coding genes, 36 tRNA genes, and 8 rRNA genes. Phylogenetic analysis revealed that *H. uninervis* shares the closest taxonomic relationship with *Cymodocea rotundata* and *Syringodium isoetifolium*, and all three species belong to the family Cymodoceaceae.

## Introduction

Seagrasses are flowering herbaceous plants that grow underwater (Kuo et al. 2018). Seagrass beds are marine ecosystems formed by seagrasses, and they are widely distributed in temperate and tropical coastal waters (Huang et al. 2006). As one of the most productive aquatic ecosystems in the biosphere (Gullström et al. 2002), seagrass beds are of great value in carbon and nitrogen sequestration due to their high productivity (Beer and Wetzel 1982; Welsh 2000). The carbon and nitrogen sequestrated can be used by herbivores such as sea turtles, sea urchins, and some marine mammals (Heck and Valentine 2006).

There have been 74 species of seagrasses recorded worldwide, belonging to 13 genera and 6 families (Short et al. 2011; Unsworth and Cullen-Unsworth 2014). The family Cymodoceaceae notably includes 18 of the 74 species, and all plants of this family are considered seagrasses (Short et al. 2011). Within this family, the genus Halodule contains 7 species, and none of the chloroplast genome sequences for these 7 species are known to date. *Halodule uninervis* (Forssk.) Boiss. 1882 is one of these species. *H. uninervis* has creeping rhizomes. The leaves are narrowly linear (McMillan, 1983), and each leaf is divided into a sheath and a blade. The leaves are 4–11 cm long and 0.8–1.4 mm wide, with three veins. Additionally, *H. uninervis* has small flowers and small round-ovate fruits (https://www.worldfloraonline.org/taxon/wfo-0000769316). *H. uninervis* is distributed from East Africa throughout the Indian Ocean to the Pacific, along coastal waters (https://www.iucnredlist.org/species/173328/6991773). It can form seagrass beds alone in some areas, or with other seagrasses (Skelton and South 2006).

Since 1980, the area of seagrass bed ecosystems has been decreasing at a rate of 110 km^2^ per year globally, approximately 7% of their total area (Waycott et al. 2009). Despite the proven importance of genetic research for successful seagrass ecosystem restoration, genetic research has often been overlooked and underutilized (Pazzaglia et al. 2021). Furthermore, the status of individual seagrass species has received little attention (Short et al. 2011).

In plant studies, the chloroplast genome has been extensively characterized at the molecular level, providing crucial information to support comparative evolutionary research. Not only is the chloroplast genome size typically small, its rate of nucleotide substitution is also relatively slow, offering an appropriate level of resolution to study plant phylogeny and evolution over relevant timescales (Clegg et al. 1994). Therefore, a complete description of molecular changes in the chloroplast genome is essential for understanding evolutionary mechanisms and developing accurate mutation models. However, the sequence, structure, and characteristics of the chloroplast genome of *H. uninervis* remained unknown. To address this, we conducted the first complete sequencing of its chloroplast genome. We characterized the *H. uninervis* chloroplast genome and analyzed its phylogenetic relationships with related species, providing novel genetic information on this species.

## Materials and methods

**Figure 1.**
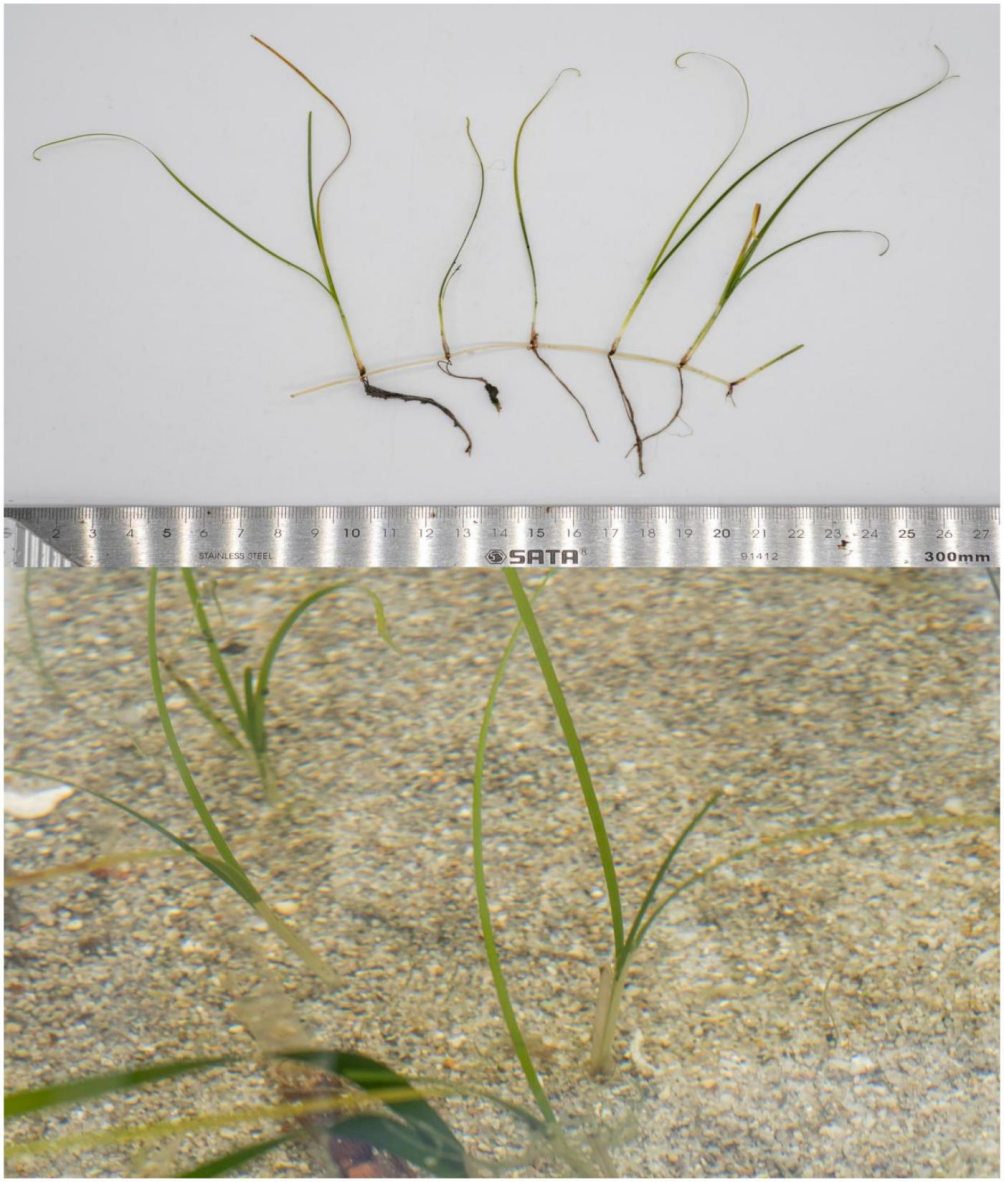
Photos of *Halodule uninervis* and the plant in its natural habitat underwater, taken in Xincun Lagoon, Hainan Island, China. The leaf blade is typically shorter than 15 cm. The leaf width ranges from 0.08 to 0.14 cm. The leaves are linear in shape and flat, with a well-developed leaf sheath and smooth leaf margins. Photo credit: Mingzhong Liu.

Fresh leaves of *H. uninervis* (Figure 1) were collected from Xincun Lagoon (18.41°N, 109.99°E), located on the southeast coastline of Hainan Island, China. The specimen was deposited in the herbarium of Biomarker Technology company (http://www.bmkgene.com/) in Qingdao, China(Contact: Jinhu Mu, mujh@biomarker.com.cn), under the voucher number 01R0048. Total genomic DNA from the fresh leaves of *H. uninervis* was extracted using the CTAB method (Doyle and Doyle 1987).

The obtained DNA was used for DNA library construction with VAHTS Universal DNA Library Prep Kit (ND607-02), and the library was sequenced on the Illumina Novaseq 6000 platform (Illumina, San Diego, CA) with paired-end reads (150 bp × 2). The complete chloroplast genome of *H. uninervis* was assembled *de novo* using SPAdes v.3.9.0 (Bankevich et al. 2012) based on the depth of coverage (Figure S1). Samtools v1.7 (Li et al. 2009) was used for the sequencing depth assessment. The protein-coding genes (PCGs), ribosomal RNA (rRNA) genes, and transfer RNA (tRNA) genes were annotated using GeSeq (Tillich et al. 2017), with manual adjustments. Genome structure annotation was performed using Geneious v.9.0.2 (Kearse et al. 2012). The sequence of the annotated genome was submitted to the GenBank database (Accession Number: OR098797). The trans-splicing and cis-splicing genes (Figures S2 & S3) of the *H. uninervis* chloroplast genome were plotted with CPGview (Liu et al. 2023). We used OGDraw (Greiner et al. 2019) (https://chlorobox.mpimp-golm.mpg.de/OGDraw.html) to visualize the genome map.

**Figure 2.**
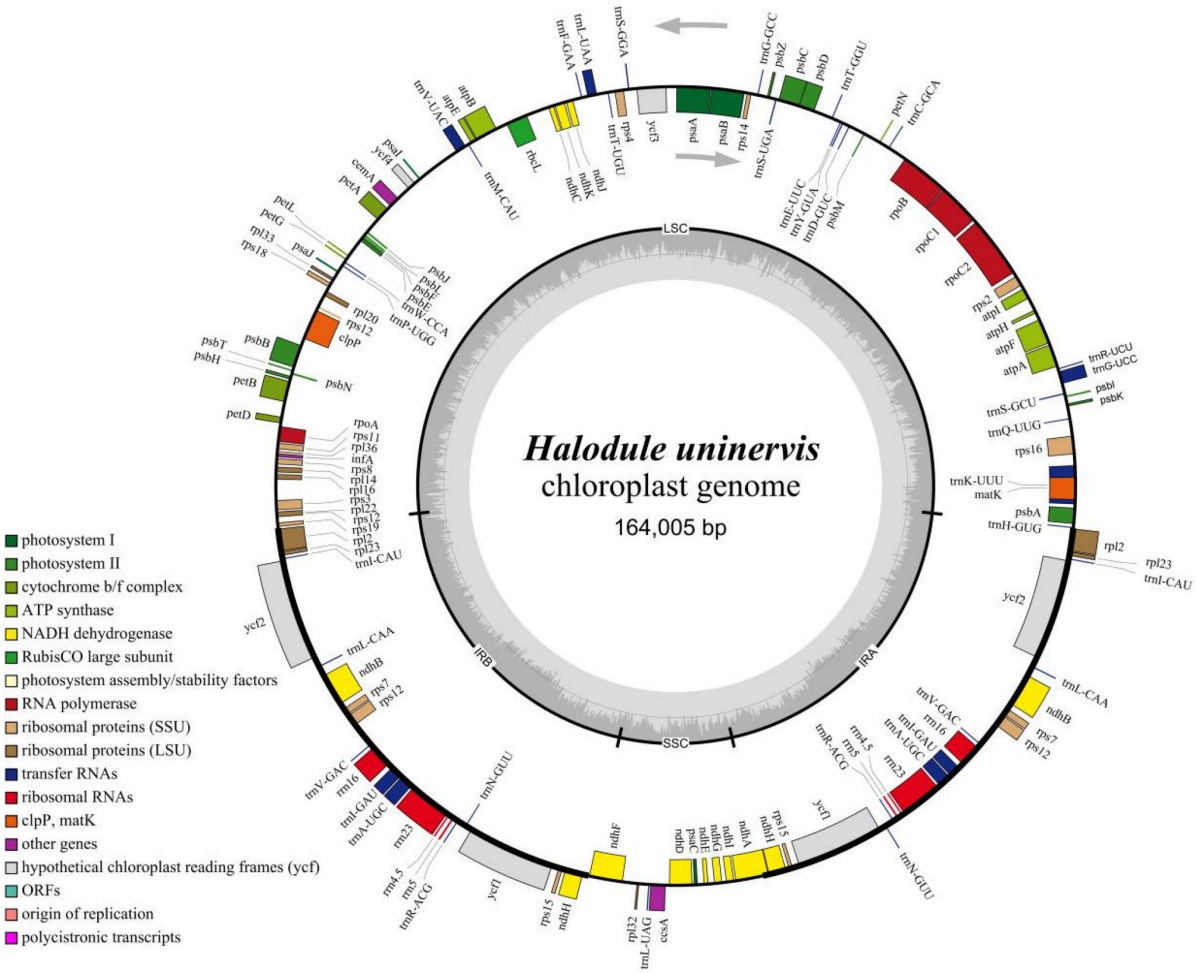
Complete chloroplast genome map of *Halodule uninervis*. The outer ring is color-coded according to gene function, and genes transcribed in clockwise and counterclockwise directions are positioned inside and outside the ring, respectively. The dark gray area inside the inner ring represents the GC content, the light gray area represents the AT content, and the gray line in the Middle indicates the 50% threshold. The quadripartite structures (LSC, SSC, IRs) are labeled on the inner ring.

**Figure 3.**
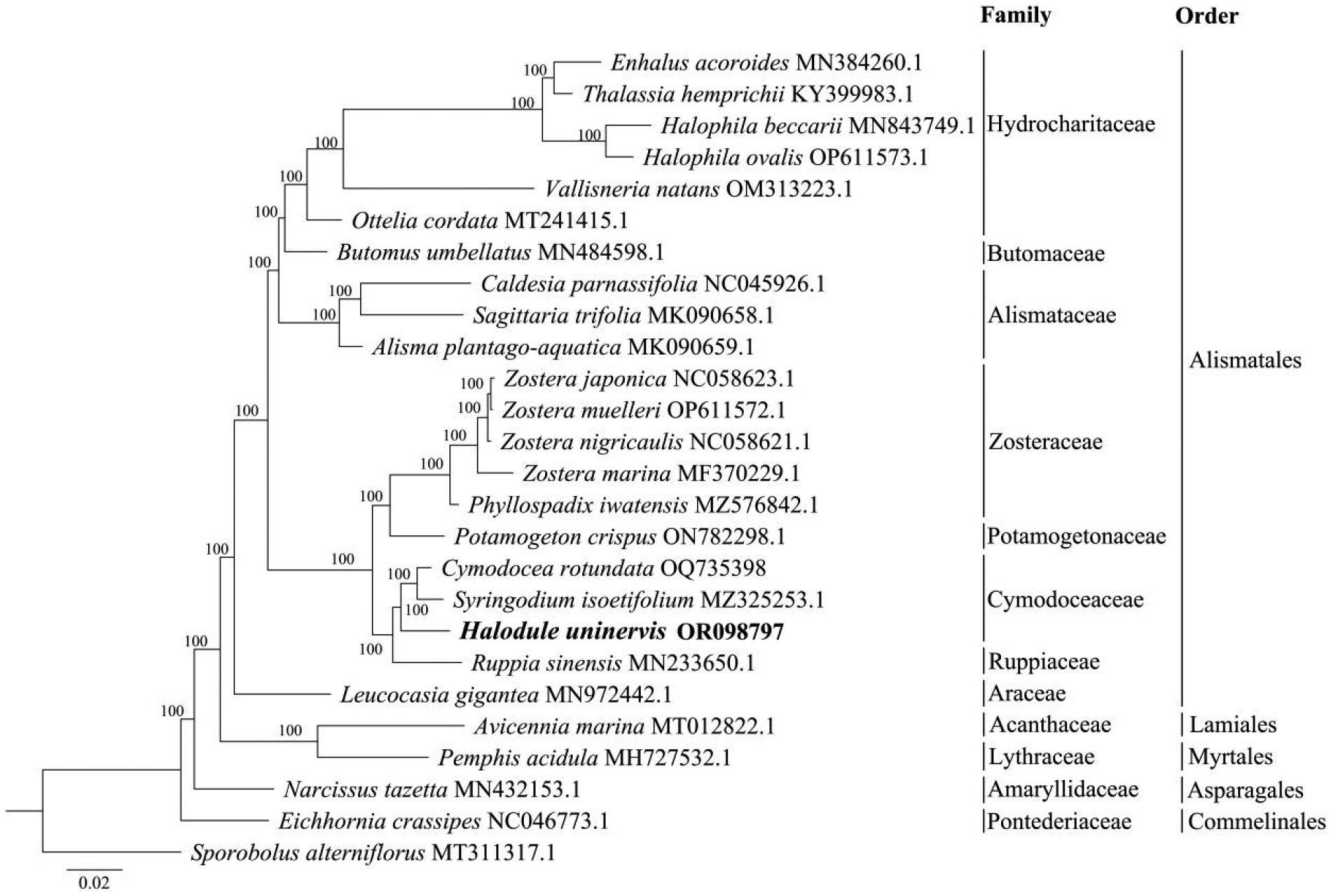
Maximum likelihood phylogenetic tree constructed based on the complete chloroplast genome sequences of *Halodule uninervis* and 24 related species with *Sporobolus alterniflorus* (MT311317.1) as the outgroup. Numbers above nodes indicate bootstrap values with 1000 replicates. The sequence of interest, *H. uninervis* (OR098797), is shown in bold. The following sequences were used: *Enhalus acoroides* MN384260.1, *Thalassia hemprichii* KY399983.1, *Halophila beccarii* MN843749.1, *Halophila ovalis* OP611573.1, *Vallisneria natans* OM313223.1, *Ottelia cordata* MT241415.1, *Butomus umbellatus* MN484598.1, *Caldesia parnassifolia* NC045926.1, *sagittaria trifolia* MK090658.1, *Alisma plantago-aquatica* MK090659.1, *Zostera japonica* NC058623.1, *Zostera muelleri* OP611572.1, *Zostera nigricaulis* NC058621.1, *Zostera marina* MF370229.1, *Phyllospadix iwatensis* MZ576842.1, *Potamogeton crispus* ON782298.1, *Cymodocea rotundata* OQ735398, *Syringodium isoetifolium* MZ325253.1, *Ruppia sinensis* MN233650.1, *Leucocasia gigantea* MN972442.1, *Avicennia marina* MT012822.1, *Pemphis acidula* MH727532.1, *Narcissus tazetta* MN432153.1, *Eichhornia crassipes* NC046773.1.

The chloroplast genomes of 24 related species, including *Sporobolus alterniflorus* as the outgroup, were chosen from the GenBank database for phylogenetic analysis. The complete chloroplast genome sequences of all the species were aligned by the MAFFT v.7 program (scoring matrix = 200, PAM *k* = 2, gap open penalty = 1.53, offset value = 0.123) (Katoh and Standley 2013). A maximum likelihood (ML) tree based on complete chloroplast genome sequences was constructed using IQ-TREE v.1.6.12 (Nguyen et al. 2015) with the ultrafast bootstrap approximation method (1000 replicates).

## Results

The chloroplast genome of *H. uninervis* is a circular molecule spanning 164,005 bp in length (Figure 2), with an average sequencing depth of 560.4× (2× to 945×) (Figure S1). It consists of a large single-copy region (LSC; 87,522 bp), a small single-copy region (SSC; 11,481 bp), and a pair of inverse repeat regions (IRs; 32,501 bp) in total. The chloroplast genome has a total GC content of 36.3%. The GC contents in the LSC, SSC, and IR regions are 34.3%, 29.4%, and 40.1%, respectively. A total of 131 genes were annotated in the *H. uninervis* chloroplast genome, including 87 PCGs, 36 tRNA genes, and 8 rRNA genes. Among the annotated genes, 8 PCGs (*ndh*A, *ndh*B, *pet*B, *atp*F, *rpl*2, *rps*16, *rpo*C1, *ycf*2) and 6 tRNA genes (*trn*A-UGC, *trn*G-UCC, *trn*I-GAU, *trn*K-UUU, *trn*L-UAA, *trn*V-UAC) contain one intron, 3 PCGs (*rps*12, *clp*P, *ycf*3) contain two introns, and all other genes contain no introns. Additionally, 9 PCGs (*ndh*B, *ndh*H, *rpl*2, *rpl*23, *rps*12, *rps*15, *rps*7, *ycf*1, *ycf*2), 7 tRNA genes (*trn*A-UGC, *trn*I-CAU, *trn*I-GAU, *trn*L-CAA, *trn*N-GUU, *trn*R-ACG, *trn*V-GAC), and all the rRNA genes are multi-copy genes with two copies, while all remaining genes are single-copy. Furthermore, only one gene (*rps*12) undergoes trans-splicing, while 13 genes (*rps*16, *atp*F, *rpo*C1, *ycf*3, *clp*P, *pet*B, *rpl*2, *ycf*2, *ndh*B, *ndh*A, *ndh*B, *ycf*2, *rpl*2) undergo cis-splicing (Figures S2 & S3).

In addition to the outgroup, *S. alterniflorus*, the complete chloroplast sequences of 24 species from 12 families and 5 orders (Alismatales, Lamiales, Myrtales, Asparagales, and Commelinales) were chosen to build the ML tree (Figure 3). All the clades on the tree were strongly supported with a bootstrap value of 100% at each node. The phylogenetic analysis revealed that *H. uninervis* was closely related to the clade formed by *Cymodocea rotundata* and *Syringodium isoetifolium*, both in the family Cymodoceaceae as well. The family Cymodoceaceae clustered with *Ruppia sinensis* from the family Ruppiaceae, and then this clade grouped with a clade consisting of species from the families Zosteraceae and Potamogetonaceae. At the order level, Lamiales and Myrtales were relatively closer to Alismatales on the tree than other orders were.

## Discussion and conclusion

The phylogenetic relationship of different seagrass species based on complete chloroplast genome sequences in this study was consistent with previous studies (Liu et al. 2019; Yu et al. 2020a, 2020b). A related study showed that *S. isoetifolium* (family Cymodoceaceae) grouped with two *Ruppia* species (family Ruppiaceae) (Ruan et al. 2022). This was before the chloroplast genome of *H. uninervis*, also from Cymodoceaceae, was sequenced. Our study revealed that *H. uninervis* is positioned between *S. isoetifolium* and *R. sinensis*, without changing the previous phylogenetic relationship of Cymodoceaceae and Ruppiaceae. On the ML tree, species in the order Alismatales diverged into eight families, and all the seagrass species on the tree belonged to this order. Within Alismatales, seagrasses and some freshwater hydrophytes, such as *Ottelia cordata*, *Butomus umbellatus*, and *Potamogeton crispus*, overlapped with each other in their phylogenetic placement, suggesting an inconsistency between chloroplast genomics characteristics and environmental adaptation.

Seagrasses are in a polyphyletic group of marine angiosperms (Olsen et al. 2016; Larkum et al. 2018). Seagrasses’ complete adaptation to marine environments as flowering plants, when compared with their terrestrial relatives, makes them highly valuable in genetic research (Olsen et al. 2016; Ma et al. 2024). Compared to nuclear and mitochondrial genomes, chloroplast genomes are highly conserved in evolution, making them particularly suitable for analyzing phylogenetic relationships over long evolutionary timescales (Daniell et al. 2016). With advances in high-throughput sequencing, studies of chloroplast genomics can address previously unexplored questions in seagrass phylogeny and comparative genomics (Chen et al. 2021). Insights into the chloroplast genome of *H. uninervis* are expected to facilitate further investigations into phylogenetic relationships and the evolution of chloroplast genomics in seagrasses.

## Supplementary Material

Supplementary Figure 3 cis splicing gene map.jpg

Supplementary Figure 1 Coverage depth.jpg

Supplementary Figure 2 trans splicing gene map.jpg

## Data Availability

The genome sequence data that support the findings of this study are openly available in GenBank of NCBI at [https://www.ncbi.nlm.nih.gov/genbank/] under the accession no. OR098797. The associated Bio-Project, SRA, and Bio-Sample numbers are PRJNA980439, SRR24833615, and SAMN35637025, respectively.
