# Supplementary figures and images for: The first complete chloroplast genome of Halodule uninervis (Forssk.) Boiss. 1882 (Cymodoceaceae)
Source: Mitochondrial DNA B Resour. 2024 Nov 20;9(11):1564–8. doi: 10.1080/23802359.2024.2429635 (PMC11580142; doi:10.1080/23802359.2024.2429635)

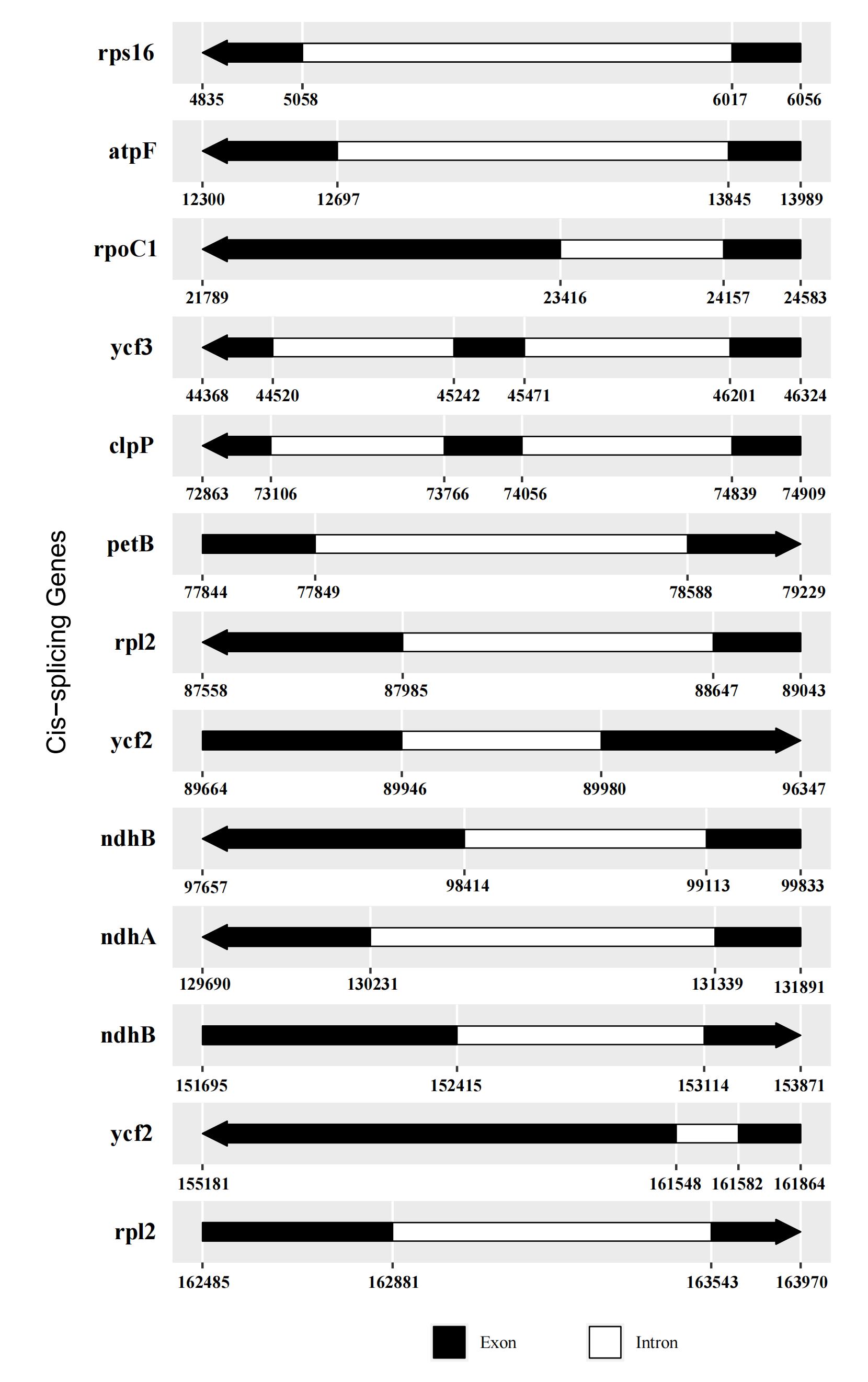

Supplement: Supplementary Figure 3 cis splicing gene map.jpg [file TMDN_A_2429635_SM7669.jpg]

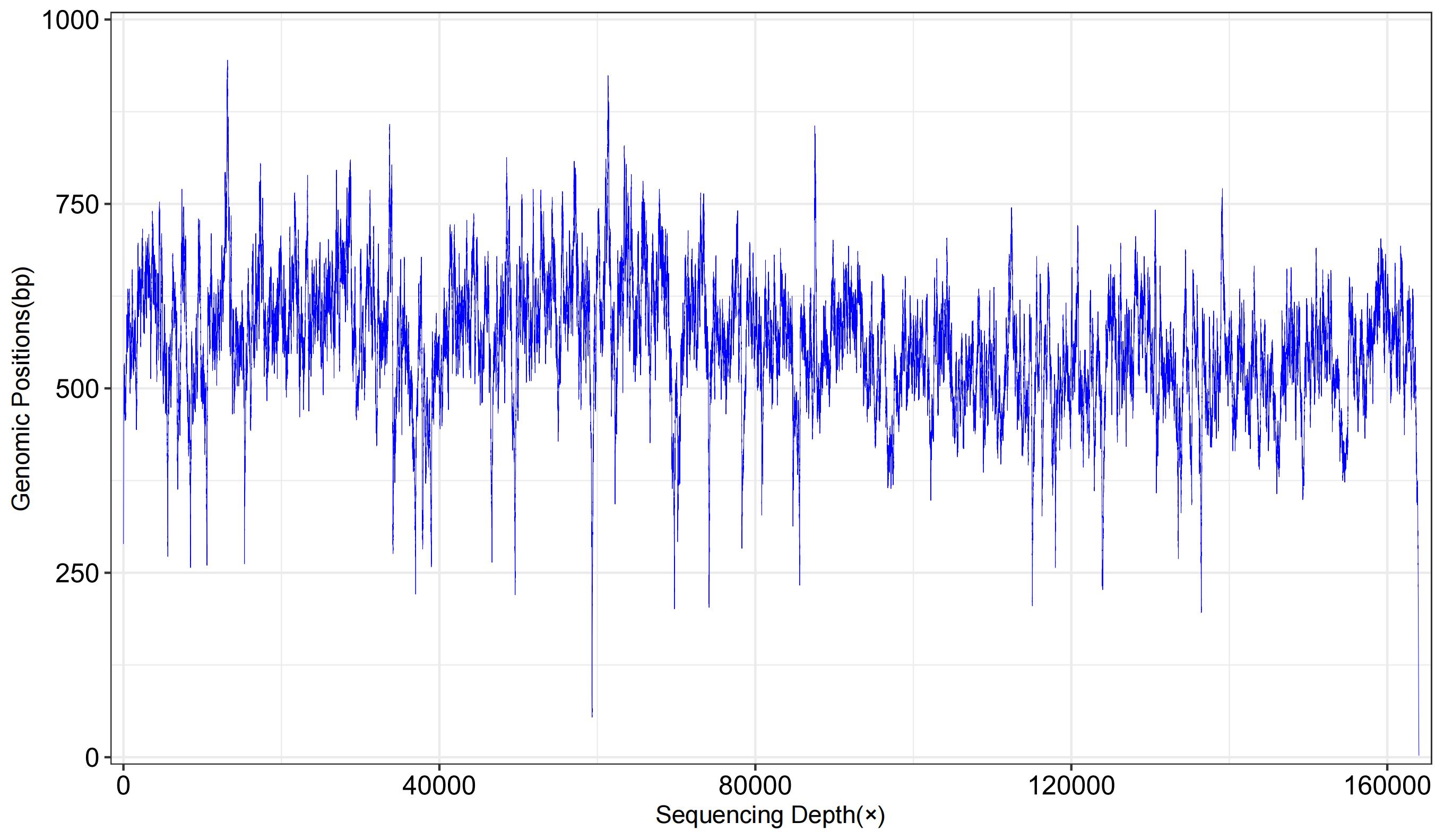

Supplement: Supplementary Figure 1 Coverage depth.jpg [file TMDN_A_2429635_SM7668.jpg]

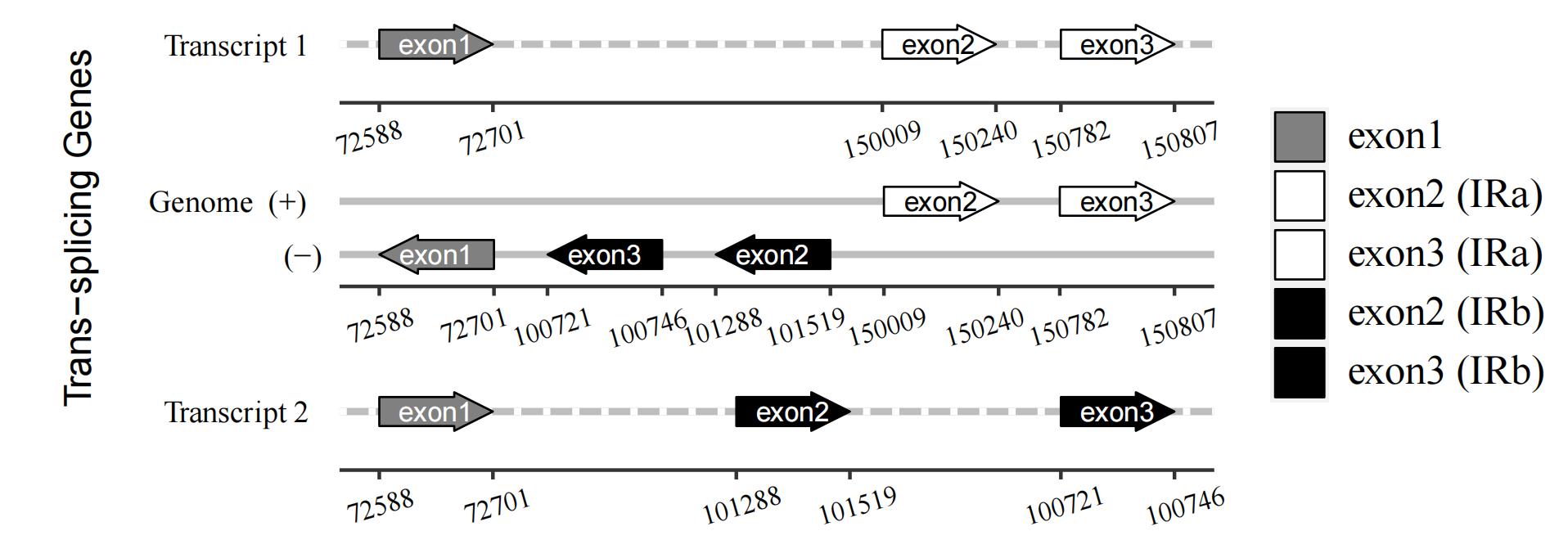

Supplement: Supplementary Figure 2 trans splicing gene map.jpg [file TMDN_A_2429635_SM7667.jpg]
